# Hepatoprotective effect of diammonium glycyrrhizinate in the treatment of pulmonary tuberculosis

**DOI:** 10.12669/pjms.40.11.10428

**Published:** 2024-12

**Authors:** Xiao Zheng, Jinjuan Xu

**Affiliations:** 1 Xiao Zheng Department of Infectious, The First People’s Hospital of Linping District, Hangzhou City, Zhejiang Province 311100, P.R. China; 2 Jinjuan Xu Department of General Geriatrics, Linping District Integrated Traditional Chinese and Western Medicine Hospital, Hangzhou City, Zhejiang Province 311100, P.R. China

**Keywords:** Diammonium glycyrrhizinate, Pulmonary tuberculosis, Hepatoprotective effect

## Abstract

**Objective:**

To explore the hepatoprotective effect of diammonium glycyrrhizinate (DG) in the treatment of pulmonary tuberculosis.

**Methods:**

This is a retrospective analysis of clinical data of 113 patients with pulmonary tuberculosis, admitted to The First People’s Hospital of Linping District from March 2020 to December 2022. Among them, 55 patients who received conventional anti-tuberculosis treatment (2HRZE/4HR) alone were assigned to the Routine group, while 58 patients who received anti-tuberculosis treatment combined with DG treatment were assigned to the DG group. Treatment efficacy, incidence of liver injury, levels of total bilirubin (TBIL), alanine aminotransferase (ALT), aspartate aminotransferase (AST), and adverse reactions were assessed in the two groups.

**Results:**

The total efficacy of treatment was significantly higher, and the incidence of liver injury was significantly lower in the DG group compared to the Routine group (*P*<0.05). After the treatment, the levels of TBIL, ALT, and AST in both groups significantly increased, and were significantly lower in the DG group than in the Routine group (*P*<0.05). There was no significant difference in the incidence of adverse reactions between the groups (*P*>0.05).

**Conclusions:**

In the treatment of pulmonary tuberculosis, combining DG with conventional anti-tuberculosis regimen can improve overall intervention effect, reduce incidence of liver injury, and alleviate its degree.

## INTRODUCTION

Pulmonary tuberculosis, caused by Mycobacterium tuberculosis, is associated with high morbidity and mortality, and is considered one of the leading causes of death from a single infectious agent.^1^-^3^ Mycobacterium tuberculosis infection is usually treated by combining pyrazinamide, isoniazid, rifampicin, and ethambutol.^4^,^5^ However, these agents are associated with some degree of liver toxicity.^6^ Research has shown that approximately 31.3% of pulmonary tuberculosis patients may experience drug-induced liver injury after the treatment.^7^,^8^ Drug-induced liver injury can affect patient treatment compliance, and cause some patients to change treatment plans or discontinue medication, ultimately affecting the overall treatment effect.^8^ Therefore, reducing or preventing liver injury is of great significance for improving treatment outcomes of patients with pulmonary tuberculosis.

Studies have shown that prophylactic hepatoprotection during anti-tuberculosis treatment can prevent the occurrence of drug induced liver toxicity and alleviate liver damage caused by anti-tuberculosis drugs.^6^-^8^ Diammonium glycyrrhizinate (DG) is a popular drug in traditional Chinese medicine that slowly metabolizes into glycyrrhetic acid^9^ which plays a role in detoxification, immune regulation, and liver cell membrane protection.^10^ While a number of studies indicated the efficiency of DG for the prevention of anti-tuberculosis drug-induced liver injury,^9^,^10^ there is still not enough evidence to support its routine prescription to prevent liver damage in people on tuberculosis treatment.

This study intends to conduct a retrospective analysis of clinical data of pulmonary tuberculosis patients in our hospital to explore the effectiveness of DG in preventing liver damage and treating pulmonary tuberculosis.

## METHODS

Retrospective selection of clinical data from 113 patients with pulmonary tuberculosis admitted to The First People’s Hospital of Linping District from March 2020 to December 2022 were analyzed. Based on the treatment received by the patients, those who received conventional anti-tuberculosis treatment were assigned to the Routine group, while those who were treated with anti-tuberculosis therapy combined with DG therapy were assigned to the DG group.

### Ethical Approval:

The ethics committee of the First People’s Hospital of Linping District approved the study, No. 2022-069, Date: June 15^th^, 2022.

### Inclusion criteria:

- Met the diagnostic criteria for pulmonary tuberculosis.^6^
- Primary pulmonary tuberculosis patients.
- Normal liver function.
- No drug allergies.
- The clinical data was complete.

### Exclusion criteria:

- Presence of significant organic lesions in organs.
- Intellectual disabilities and mental system disorders.
- Patients with diabetes.
- Individuals with immune system disorders.
- Breastfeeding and pregnant women.

### Routine group:

Patients received standard anti-tuberculosis treatment 2HRZE/4HR, namely two months of isoniazid (H), rifampicin (R), pyrazinamide (Z) and ethambutol (E), followed by continuation phase of four months of isoniazid and rifampicin. Patients received administration of ethambutol hydrochloride (Hangzhou Minsheng Pharmaceutical Co., Ltd.; specification: 0.25 g/tablet; approval number: H33021602), once a day, 0.75 g/time; Intravenous infusion of rifampicin (Shenyang Shuangding Pharmaceutical Co., Ltd.; specification 5ml: 0.3g; approval number: H20050725), once a day, 0.45 g/time; Oral pyrazinamide tablets (Suzhou Homesun Pharmaceutical Co., Ltd.; specification: 0.25g/tablet; approval number: H32024490), once a day, 1.5 g/time; Oral isoniazid (Shanghai Xinyi Yellow River Pharmaceutical Co., Ltd.; specification: 100 mg/tablet; approval number: H31020495), once a day, 0.3 g/time.

### DG group:

In addition to the standard drug regimen (see above), prophylactic hepatoprotection treatment was administered using DG enteric coated capsules (produced by Zhengda Tianqing Pharmaceutical Group Co., Ltd.; specification: 50mg per capsule; approval number: H20040628), orally, taken three times a day, 150 mg/time. Continuous treatment lasted for six months.

### Observation indicators:

- Treatment effect. The absence of active lesions in lung examination indicated clinical cure; The absorption of lung lesions ≥ 80% was considered significant; A lung lesion absorption rate of 30% to 79% was considered effective; If the absorption rate of lung lesions was ≤ 29%, it was considered invalid; Effective, significant, and clinically cured were included in the total effective rate.
- The occurrence of liver injury was determined based on the assessment of alanine aminotransferase (ALT) levels. Based on the upper limit of normal (ULN), severity of liver injury was classified into mild (< 2 × ULN), moderate (2-5 × ULN), and severe (>5 × ULN).
- Liver function indicators, such as serum levels of total bilirubin (TBIL), ALT, and aspartate aminotransferase (AST), were measured by enzyme-linked immunosorbent assay (ELISA) using the RT-6100 developed by Beckman Coulter in the United States. The reagent kit was purchased from BOSTER Biological Technology co. ltd.
- The incidence of adverse reactions, including vomiting, nausea, decreased appetite, fatigue, and increased blood pressure.

### Statistical Analysis:

All data analyses were conducted using SPSS 25.0 software (IBM Corp, Armonk, NY, USA). Quantitative data were shown as mean ± standard deviation, independent sample t-test was used for intergroup comparison, and paired t-test was used for intra group before and after comparison. Count data were shown as n (%) and analyzed using Chi-square test. When P<0.05, the difference was considered statistically significant.

## RESULTS

This study included a total of 113 patients (59 males and 54 females), aged from 29 to 72 years, with a mean age of 52.49 ± 11.19 years. There were 58 cases in the DG group, and 55 cases in the Routine group, with no significant difference in baseline data between the two groups of patients (*P*>0.05), Table-I. The total efficacy of treatment in the DG group (91.38%) was higher than that in the Routine group (76.36%) (*P*<0.05), Table-II.

**Table-I:** Comparison of baseline data between two groups.

Group	Gender (male/female)	Age (year)	BMI (kg/m²)	Type of pulmonary tuberculosis		

				Chronic fibrous cavity type	Infiltration	Tuberculous exudative pleurisy
DG group(n=58)	33/25	51.52±10.70	23.29±3.38	17 (29.31)	32 (55.17)	9 (15.52)
Routine Group (n=55)	26/29	53.51±11.70	22.49±3.08	12 (21.82)	36 (65.45)	7 (12.73)
*χ^2^/t*	1.048	-0.945	1.309	1.269		
*P*	0.306	0.347	0.193	0.530		

**Table-II:** Comparison of treatment effects between two groups.

Group	n	Clinically cured	Significant	Effective	Invalid	Total effective rate
DG group	58	28 (48.28)	16 (27.59)	9 (15.51)	5 (8.62)	53 (91.38)
Routine group	55	17 (30.91)	11 (20.00)	20 (36.36)	7 (12.73)	48 (87.27)
*χ^2^*						8.047
*P*						0.045

**Fig.1:**
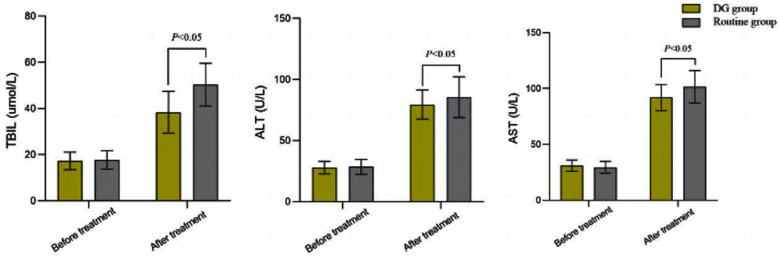
Comparison of liver function indicators between two groups.

The incidence of liver injury in the DG group (18.97%) was lower compared to the Routine group (40.00%) (*P*<0.05) Table-III. Before the treatment, there was no significant difference in TBIL, ALT, and AST levels between the two groups (*P*>0.05). After the treatment, levels of TBIL, ALT and AST in both groups increased, and were significantly lower in the group compared to the Routine group (*P*<0.05), Fig.1. There was no significant difference (*P*>0.05) in the incidence of adverse reactions between the DG (8.62%) and the Routine group (3.64%), Table-IV.

**Table-III:** Comparison of liver injury occurrence between two groups.

Group	n	Mild injury	Moderate injury	Severe injury	Total incidence rate
DG group	58	7 (12.07)	2 (3.45)	2 (3.45)	11 (18.97)
Routine group	55	7 (12.73)	11 (20.00)	4 (7.27)	22 (40.00)
*χ^2^*					9.274
*P*					0.026

**Table-IV:** Comparison of incidence rates of adverse reactions between two groups.

Group	n	Vomiting and nausea	Loss of appetite	Fatigue	Increased blood pressure	Total incidence rate
DG group	58	2 (3.45)	2 (3.45)	0 (0.00)	1 (1.72)	5 (8.62)
Routine group	55	0 (0.00)	1 (1.82)	1 (1.82)	0 (0.00)	2 (3.64)
*χ*^2^Yates						0.502
*P*						0.479

## DISCUSSION

Our study showed that DG regiment combined with the conventional anti-tuberculosis treatment is safe and is associated with higher efficacy (91.38%) compared to the conventional anti-tuberculosis regiment (76.36%) (P<0.05). It has been showed that DG can effectively reduce liver function indicators such as ALT, AST, and TBIL, and has a certain effect on liver function recovery. The results of a randomized controlled trial by Lin et al.^11^ also showed that combining anti-tuberculosis regimens with DG for prophylactic liver protection treatment is beneficial for improving the therapeutic effect of primary diseases. Our results are in line with previous reports.^9^,^11^ We may speculate that DG has certain detoxification, anti-inflammatory, and immune regulatory effects, which can help improve the effectiveness of disease treatment.^9^-^11^

Research has shown that the combination therapy of ethambutol, rifampicin, pyrazinamide, and isoniazid can kill Mycobacterium tuberculosis through different mechanisms, ensuring the effectiveness of pulmonary tuberculosis treatment.^5^,^12^ However, these drugs can have a strong stimulating effect on the liver, which can easily cause an increase in transaminases and damage liver function.^12^ This study analyzed the extent of liver injury of pulmonary tuberculosis patients before and after the treatment, and showed that the incidence of liver injury in the DG group (18.97%) was lower than that in the Routine group (40.00%).

At the same time, levels of liver function indicators in the DG group were significantly lower than those in the Routine group, indicating that the prophylactic DG administration during routine treatment for pulmonary tuberculosis can help protect liver function and reduce the risk of liver injury.^13^ The research results of Wei et al.^13^ showed that DG can reduce the risk of drug-induced liver injury caused by anti-tuberculosis drugs, shorten the time for improving liver function related indicators, with no increase in the incidence of adverse effects. Wang et al.^14^ conducted a cost-benefit analysis of drug-induced liver injury in patients treated with anti-tuberculosis drugs, and showed that reduced glutathione, DG, and compound glycyrrhizin injection were all effective in treating drug-induced liver injury patients caused by anti-tuberculosis treatment, which may be related to the reduction of oxidative stress and inflammatory damage.

In addition, the research results of Jiang et al.^15^ indicated that the combination of standard anti-tuberculosis regimen and DG preventive liver protection treatment can effectively reduce the occurrence of liver injury. The change of anti-tuberculosis intervention plan caused by drug-induced liver injury was only 3.2%, significantly lower than 7.8% without preventive liver protection treatment. Our results further confirm observations that DG preventive liver protection therapy is feasible and effective, and can effectively protect liver function of pulmonary tuberculosis patients treated with anti-tuberculosis drugs.^13^-^15^

We may suggest that the effect of DG may be attributed to its hepatoprotective qualities, and ability to improve liver function, stabilize liver cell membrane, and anti-inflammatory activity. DG can prevent liver damage caused by hepatotoxic drugs, increase nucleic acid and liver glycogen content, and promote liver cell function recovery.^16^,^17^ In addition, studies have shown that DG can inhibit the activity of cytochrome P450 group of enzymes (namely, CYP2E1, CYP3A, and CYP1E1) in the liver, and prevent NF-kB activation, thus exerting a protective effect on liver function.^18^ DG, an ammonium salt preparation of 18-α glycyrrhizin, includes glycyrrhetinic acid and phospholipid components with low molecular polarity and high lipid solubility that can enhance the absorption rate of 18-α glycyrrhetinic acid in the small intestine, further increasing bioavailability, and protecting liver function.^19^,^20^ Additionally, lipid peroxidation products can have a stimulating effect on collagen gene transcription, leading to fibrosis.^21^ DG has strong anti-peroxidation activity and therefore, may prevent liver fibrosis.^18^-^20^

### Limitation:

Firstly, it is a single center retrospective study. The sample size is small and there is a risk of selection bias. Secondly, there are few observation indicators and no analysis of follow-up results. Finally, the impact of the two methods on the long-term functional recovery of patients was not analyzed. Further higher-quality studies are needed to verify our conclusions.

## CONCLUSION

In the treatment of pulmonary tuberculosis, combining DG with conventional anti-tuberculosis regimen can improve the overall outcome of the therapy, reduce incidence of liver injury and alleviate its degree, and is not associated with increased risk of adverse reactions.

### Authors’ contributions:

**XZ** and **JX:** Collected the data and performed the analysis, Critical Review

**XZ:** Conceived and designed the study and involved in the writing of the manuscript.

All authors have read, approved the final manuscript and are responsible for the integrity of the study.
